# High‐dose antipsychotic drug use as a predictor for readmission of inpatients with borderline personality disorder: A retrospective chart review in a Japanese psychiatric hospital

**DOI:** 10.1002/npr2.12140

**Published:** 2020-10-10

**Authors:** Yuji Yamada, Yuma Yokoi, Zui Narita, Naotsugu Hirabayashi

**Affiliations:** ^1^ Department of Psychiatry National Center Hospital National Center of Neurology and Psychiatry Kodaira Japan; ^2^ Department of Mental Health Johns Hopkins Bloomberg School of Public Health Baltimore Maryland USA

**Keywords:** antipsychotics, hospitalization, personality disorder, pharmacotherapy, readmission

## Abstract

**Objective:**

This study aimed to determine predictors associated with readmission of inpatients with borderline personality disorder.

**Methods:**

This observational study evaluated 83 inpatients with borderline personality disorder admitted to the National Center of Neurology and Psychiatry Hospital in Japan from January 2013 to January 2016. Data were retrospectively obtained from electronic medical records.

**Results:**

There was no significant difference in the daily antipsychotic dose equivalent to chlorpromazine at admission between the readmitted and nonreadmitted groups, which indicated that there was no between‐group difference in the psychiatric disease severity at admission. Multivariate logistic regression analyses revealed that the use of antipsychotics equivalent to >400 mg of chlorpromazine at discharge was associated with readmission within 1 year.

**Conclusions:**

In conclusion, high‐dose antipsychotic drug use at discharge may be a risk factor for readmission. The present findings may have important clinical implications since they alert physicians to a possible predictor for readmissions of patients with borderline personality disorder.

## Abstract 2

There is limited available information regarding predictors associated with repetitive admission of patients with BPD. This study aimed to evaluate predictors associated with readmission of inpatients with BPD. 1. Multivariate logistic regression analyses revealed that the use of antipsychotics equivalent to >400 mg of chlorpromazine at discharge was associated with readmission within 1 year. 2. High‐dose antipsychotic drug use at discharge may be a risk factor for readmissions.

## Graphical abstract

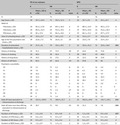

**Key points**

- There is limited available information regarding predictors associated with repetitive admission of patients with BPD. This study aimed to evaluate predictors associated with readmission of inpatients with BPD.
- Multivariate logistic regression analyses revealed that the use of antipsychotics equivalent to >400 mg of chlorpromazine at discharge was associated with readmission within 1 year.
- High‐dose antipsychotic drug use at discharge may be a risk factor for readmissions.

## 1 INTRODUCTION

Borderline personality disorder (BPD) is a common disease in psychiatric settings with a morbidity of approximately 9.1% in adults.^1^ Patients with BPD are often hospitalized with those with severe BPD being repeatedly admitted.^2^ Previous studies have suggested that a longer inpatient treatment duration is not necessarily effective for suicide prevention^3^; moreover, the hospitalization duration should be as short as possible.^4^ This is because hospitalization is occasionally counter‐therapeutic for patients with BPD since they tend to be regressive through this practice^3^; further, their symptoms are often severe and treatment‐resistant.^5^ Additionally, patients with BPD tend to repeatedly use psychiatric services and consume social resources.^6^ Therefore, there is a need for evidence‐based strategies for minimizing hospitalization.

Several studies have examined predictors associated with hospitalization in patients with BPD. Pascual et al (2007) reported that the risk of suicide, danger to others, and symptom severity were associated with hospitalization in patients with BPD.^7^ Paolini et al (2017) reported that prescribing antipsychotic and/or antidepressant medication increased the length of hospitalization; contrastingly, mood stabilizer usage reduced it.^8^ However, there is little available information regarding the predictors associated with readmission of patients with BPD. Therefore, this study aimed to evaluate predictors associated with readmission of inpatients with BPD.

## 2 METHODS

This study was approved by the Clinical Research Ethics Committee of the National Center of Neurology and Psychiatry Hospital (A2017‐039).

### 2.1 Study design and data sources

We evaluated 83 inpatients diagnosed with BPD based on the ICD‐10 Classification of Mental and Behavioral Disorders in the National Center of Neurology and Psychiatry in Japan from January 2013 to January 2016 (see Table S1). The requirement for informed consent was waived, and an opt‐out option was availed since the data were retrospectively obtained from electronic medical records.

### 2.2 Study variables

The study variables included the following: age, sex, family history of psychiatric disorder, education level, job career, history of alcohol drinking, history of smoking, history of drug abuse, self‐harm during hospitalization, Wechsler Adult Intelligence Scale scores (full scale, verbal, and performance IQ [intelligence quotient]), diagnosis on admission, psychiatric comorbidity, age at first‐visit of a psychiatric hospital, hospitalization duration, duration of preceding hospitalization, readmission within 1 year, and prescribed drugs at admission and discharge. We classified prescribed drugs into the following four categories: antipsychotics, mood stabilizers, antidepressants, and benzodiazepines. Furthermore, the equivalent doses of chlorpromazine, diazepam, and imipramine at admission and discharge were calculated (see Table S2).^9^

### 2.3 Statistical analysis

Data were analyzed using the Stata 14 software package (Stata Corp. 2015. Stata Statistical Software: Release 14. College Station, TX, Stata Corp LP). Predictors associated with readmission were examined using multivariate logistic regression analyses. Variables with *P* < .20 in the univariate logistic regression analyses were included in the multivariate logistic regression model. Additionally, the study sample was grouped as follows: the readmitted and nonreadmitted groups. Between‐group differences in demographic characteristics were examined using independent‐sample *t* tests or Fisher's exact tests.

## 3 RESULTS

Table 1 summarizes the demographic features of the inpatients. There were 110 and 83 inpatients with personality disorder (PD) and BPD, respectively (see Table 1 and Table S1).^10^ Among the inpatients with BPD, 32 (38.6%) individuals were readmitted to hospital within 1 year (see Table 1). Moreover, there was no significant between‐group difference in the daily antipsychotic doses equivalent to chlorpromazine at admission (readmitted group: max = 1200, median = 68; nonreadmitted group: max = 1132.7, median = 43), which indicated no between‐group difference in the psychiatric disease severity at admission. Independent‐sample *t* tests and Fisher's exact tests revealed significant between‐group differences among patients with BPD in the duration of antecedent hospitalization (*P* = .001) and daily antipsychotic dose >400 mg equivalent to chlorpromazine at discharge (*P* = .005) (Table 1). Contrastingly, there were no significant between‐group differences among the patients with PDs included other than BPD (see Table 1). During hospitalization, benzodiazepines were the most frequently prescribed drugs, followed by antipsychotics, mood stabilizers, and antidepressants (see Table 2). Further, data about psychosocial interventions were not obtained from the electronic medical records, as it was difficult to quantitatively or qualitatively evaluate them.

**TABLE 1:** Background information regarding the readmitted and nonreadmitted groups

	PD of any subtypes					BPD				
	R		NR		*P* value	R		NR		*P* value
	N	Mean ± SD or n (%)	N	Mean ± SD or n (%)		N	Mean ± SD or n (%)	N	Mean ± SD or n (%)	
N	39		71			32		51		
Age (mean ± SD)	39	35.7 ± 10.3	71	35.9 ± 11.5	.9	32	34.7 ± 9.1	51	33.1 ± 8.7	.4
WAIS‐III										
FIQ (mean ± SD)	24	85.2 ± 17.8	34	89.1 ± 18.3	.4	19	88.4 ± 14.9	23	89.2 ± 17.3	.8
VIQ (mean ± SD)	24	89.3 ± 16.3	34	93.2 ± 19.1	.4	19	90.9 ± 14.0	22	92.6 ± 18.3	.7
PIQ (mean ± SD)	24	82 ± 17.6	34	86.4 ± 18.1	.4	19	86.1 ± 15.1	22	87.7 ± 17.3	.7
Years of schooling (mean ± SD)	39	13.6 ± 2.7	70	13.1 ± 2.3	.3	32	13.6 ± 2.7	51	13.2 ± 2.2	.4
Age at the first psychiatric visit (mean ± SD)	39	22.8 ± 7.2	70	26.8 ± 11	.05	32	23.0 ± 7.0	51	25.9 ± 8.5	.1
Duration of antecedent hospitalization (mean ± SD)	39	31.9 ± 41	70	19.6 ± 59.1	.3	32	32.0 ± 41.3	51	10.0 ± 18.0	**.001**
History of drinking	33	51.5	57	71.9	.05	27	63.0	42	73.8	.3
History of smoking	35	48.5	58	46.5	.9	28	50.0	43	48.8	.9
History of drug abuse	29	10.3	51	17.6	.4	24	12.5	38	15.8	.7
History of self‐harm	39	84.6	69	66.6	.04	32	93.8	50	86	.2
Psychiatric comorbidity										
F0	39	5.1	71	4.2	.8	32	0	51	2.0	.4
F1	39	7.6	71	4.2	.5	32	6.3	51	5.9	.9
F2	39	10.2	71	11.2	.9	32	6.3	51	5.9	.9
F3	39	15.3	71	28.1	.1	32	18.8	51	33.3	.1
F4	39	28.2	71	15	.1	32	28.1	51	15.7	.1
F5	39	0	71	2.8	.3	32	0	51	2.0	.4
F6	39	0	71	0	0	32	0	51	0	0
F7	39	2.5	71	1.4	.67	32	3.1	51	0	.2
F8	39	0	71	0	0	32	0	51	0	0
F9	39	0	71	1.4	.5	32	0	51	2.0	.4
Daily AP dose equivalent to chlorpromazine at discharge	39	313.2 ± 109.4	71	186.9 ± 51.7	.2	32	302.0 ± 69.0	51	135.7 ± 30.2	**.014**
Daily AP dose more than 400 mg equivalent to chlorpromazine at discharge	39	30.7	71	9.8	.01	32	31.2	51	7.8	**.005**
Numbers of AP (mean ± SD)	39	1.2 ± 0.8	71	0.9 ± 0.8	.2	32	1.3 ± 0.8	51	0.9 ± 0.8	**.037**
Numbers of STB (mean ± SD)	39	0.6 ± 0.6	71	0.4 ± 0.7	.2	32	0.6 ± 0.6	51	0.4 ± 0.6	.2
Numbers of AD (mean ± SD)	39	0.5 ± 0.7	71	0.4 ± 0.6	.3	32	0.6 ± 0.7	51	0.5 ± 0.6	.4
Numbers of BDZ (mean ± SD)	39	1.2 ± 0.9	71	1.2 ± 1.1	.3	32	1.2 ± 0.9	51	1.2 ± 1.1	.9

Note

We summarized the demographic features of the inpatients and divided the included individuals into the readmitted and nonreadmitted groups. Between‐group differences in demographic features were examined using independent‐sample *t* tests or Fisher's exact tests. Bold value indicates less than .05.

Abbreviations: 1st discharge, we defined the first hospitalization during the study period as the first time; AD, antidepressant; AP, antipsychotic; BDZ, benzodiazepine; BPD, borderline personality disorder; F0, organic disorders including symptomatic, mental disorders; F1, mental and behavioral disorders due to psychoactive substance use; F2, schizophrenia, schizotypal, and delusional disorders; F3, mood [affective] disorders; F4, neurotic, stress‐related, and somatoform disorders; F5, behavioral syndromes associated with physiological disturbances and physical factors; F6, disorders of adult personality and behavior; F7, mental retardation; F8, disorders of psychological development; F9, behavioral and emotional disorders with childhood and adolescence onset; FIQ, full‐scale intelligence quotient; N, number; NR, nonreadmitted group; PD, personality disorder; PIQ, performance IQ; R, readmitted group; SD, standard deviation; STB, mood stabilizer; VIQ, verbal IQ.

**TABLE 2:** Classification of drugs prescribed to patients with personality disorder (PD) upon hospital admission

	Average daily dose equivalent to reference drugs (mg/d)	Minimum dose (mg/d)	Maximum dose (mg/d)	Prevalence N (%)
AP	329.2	25	1200	67 (60.9)
STB	‐	‐	‐	49 (44.6)
AD	118.7	18.8	300	46 (41.8)
BDZ	18.4	1.5	90	70 (63.6)

Note

Antipsychotics, antidepressants, and benzodiazepines were converted into equivalent chlorpromazine, imipramine, and diazepam doses, respectively.

APs, ADs, and BDZs are converted into an equivalent chlorpromazine, imipramine, and diazepam dose, respectively.^9^

Abbreviations: AD, antidepressant; AP, antipsychotic; BDZ, benzodiazepine; N, number; PD, personality disorder; STB, mood stabilizer.

Multivariate logistic regression analyses confirmed that the use of antipsychotics equivalent to >400 mg of chlorpromazine at discharge increased the odds of readmission within 1 year (see Table 3).

**TABLE 3:** Correlation between readmission and use of antipsychotics equivalent to >400 mg of chlorpromazine in the inpatients with borderline personality disorder

	Odds ratio	Standard error	*z*	*P* > |*z*|	[95% Conf. interval]
Sex	0.36	0.34	−1.08	0.28	0.06, 2.27
History of self‐harm	1.37	1.30	0.33	0.74	0.21, 8.85
AD‐type at 1st discharge	1.40	0.54	0.87	0.38	0.66, 2.96
Daily BDZ dose equivalent to diazepam at 1st discharge	1.03	0.02	1.54	0.12	0.99, 1.06
Daily AP dose >400 mg equivalent to chlorpromazine at 1st discharge	4.74	3.18	2.32	**0.02**	1.27, 17.68
Duration of preceding hospitalization >30 d	0.86	0.47	−0.28	0.78	0.29, 2.53
Constant	0.27	0.27	−1.33	0.18	0.04, 1.86

Note

BDZs and APs are converted into equivalent diazepam and chlorpromazine doses, respectively.^9^

Abbreviations: AD, antidepressant; AD‐type at 1st discharge, number of antidepressants (ADs) used at discharge after the hospital during the first hospitalization; AP, antipsychotic; BDZ, benzodiazepine; BPD, borderline personality disorder; Conf. Interval, confidence interval; History of self‐harm, yes is “1” and no is “0”; Sex, male sex is “1” and female sex is “0”.

## 4 DISCUSSION

This study examined predictors associated with readmission of inpatients with BPD. Since the hospitalization duration should be as short as possible^4^ due to its poor effectiveness^3^, ^5^ and economic burden,^6^ our findings may have important implications for future efforts of reducing the hospitalization frequency and duration among patients with BPD.

To our knowledge, this is the first study to identify a predictor of increased readmission for patients with BPD. Using the daily antipsychotic dose equivalent to chlorpromazine at admission, we estimated the approximate severity of psychiatric diseases at admission in the readmitted and nonreadmitted groups. Multivariate logistic regression analyses revealed that the use of antipsychotics equivalent to >400 mg of chlorpromazine at discharge was associated with increased readmission within 1 year (see Table 3).

The method used to determine the threshold dose of 400 mg of chlorpromazine was based on the studies by Nickel et al (2006)^11^ and Black et al (2014).^12^ A previous study reported that 15 mg/d of aripiprazole equivalent to 375 mg of chlorpromazine was effective for treating several symptom domains of BPD; specifically, depression, anxiety, and anger.^11^ Moreover, 150 mg/d of quetiapine equivalent to 227 mg of chlorpromazine was found to significantly reduce the severity of BPD symptoms.^12^ Therefore, the present study tested the effectiveness of a higher antipsychotic dose equivalent to >400 mg of chlorpromazine.

Although the mechanism underlying BPD treatment using antipsychotics remains unclear, second‐generation antipsychotics are commonly prescribed in BPD patients.^13^ While RCTs of antipsychotics have shown mixed results,^14^ aripiprazole and quetiapine may improve instability of affect regulation, impulse control, or aggression.^11^, ^15^ The use of high‐dose antipsychotics suggests urgent need for severe symptoms such as anger/aggression, impulsivity, or paranoia during the hospitalization. Moreover, high‐dose antipsychotics have adverse effects, including sedation, change in appetite, dry mouth, and dizziness, which modifies their feeling of symptoms and thus make them more symptomatic.^12^ Therefore, a high‐dose antipsychotic drug use may work for a short while but eventually be a risk for readmission, although this should be carefully interpreted since the antipsychotic dose may merely reflect the severity of the psychiatric conditions.

Hospital admission is a clinical crisis, and avoiding readmission is a soft outcome that indicates therapeutic success in terms of patients' quality of life and proper use of social resources. However, there remains no established therapy for improving core symptom domains of BPD^13^, ^16^; therefore, pharmacotherapy is reluctantly selected against the suggestions of clinical guidelines.^17^, ^18^ Zanarini et al (2004) reported that 78% of patients with BPD were on psychotropic drugs; among them, 37% were on ≥3 psychotropics,^19^ which is consistent with our results. This could be attributed to several difficulties in showing consistent therapeutic effectiveness.^20^ First, BPD is strongly associated with other mental disorders,^19^, ^21^ which makes it difficult to distinguish symptom improvement from personality change. Second, there is no indurated quantitative evaluation of the essential BPD features. Therefore, there is little information regarding whether these core diagnosis domains are significantly improved by treatment. Taken together, there is limited evidence regarding therapy for BPD.

This study has several limitations. First, the included patients may have had more severe symptoms compared to those of the general psychiatric inpatient population. This is because the National Center of Neurology and Psychiatry, which is among the National Centers for Advanced and Specialized Medical Care in Japan, tends to hospitalize patients whose care and management are deemed too difficult for other hospitals. This suggests that caution should be exercised in generalizing these results to the whole population. Second, this study included a relatively small sample size, which may have compromised the ability to detect statistical significance and resulted in wide confidence intervals for some point estimates. Multicenter studies with larger samples and quantitative outcome measures are needed to confirm the validity of the findings.

In conclusion, the use of high‐dose antipsychotic drug use at discharge may be a risk factor for readmissions. Our findings may have important clinical implications since they alert physicians to possible predictors for readmissions of inpatients with BPD.

## CONFLICT OF INTEREST

The authors declare no competing interests.

## AUTHOR CONTRIBUTIONS

Yuji Yamada and Yuma Yokoi planned and designed the study. Yuji Yamada collected the data and drafted the first manuscript. Yuma Yokoi and Zui Narita analyzed the data. Yuma Yokoi, Zui Narita, and Naotsugu Hirabayashi critically reviewed the draft and revised it. All authors made substantial contributions and approved the final manuscript.

## APPROVAL OF THE RESEARCH PROTOCOL BY AN INSTITUTIONAL REVIEWER BOARD

This study was approved by the Clinical Research Ethics Committee of the National Center of Neurology and Psychiatry Hospital (A2017‐039).

## INFORMED CONSENT

The requirement for informed consent was waived, and an opt‐out option was availed since the data were retrospectively obtained from electronic medical records.

## Supporting information

Table S1‐S2

## Data Availability

The raw data belonged to the present study cannot be made publicly available, because the disclosure of personal data was not included in the research protocol of the present study.
