# Supplementary material for: High‐dose antipsychotic drug use as a predictor for readmission of inpatients with borderline personality disorder: A retrospective chart review in a Japanese psychiatric hospital
Source: Neuropsychopharmacol Rep. 2020 Oct 10;40(4):365–70. doi: 10.1002/npr2.12140 (PMC7722642; doi:10.1002/npr2.12140)
Supplement: Supplementary file 1 — Table S1‐S2 [file NPR2-40-365-s001.docx]

**Supplementary Table 1. Classification of diagnosis about personality disorder and psychiatric comorbidity.**

| Diagnosis | Schizoid | Paranoid | Borderline | Antisocial | Narcissistic | Avoidant | Dependent | Organic | Unknown | Total |
| --- | --- | --- | --- | --- | --- | --- | --- | --- | --- | --- |
| Persons | 2 | 6 | 83 | 3 | 1 | 4 | 1 | 9 | 1 | 110 |
| Frequency (%) | 1.8 | 5.5 | 75.5 | 2.7 | 0.9 | 3.6 | 0.9 | 8.2 | 0.9 | 100 |

PD: personality disorder, schizoid: schizoid PD, paranoid: paranoid PD, borderline: borderline PD, antisocial: antisocial PD, narcissistic: narcissistic PD, avoidant: avoidant PD, dependent: dependent PD, organic: organic PD.

| Psychiatric comorbidity | F0 | F1 | F2 | F3 | F4 | F5 | F6 | F7 | F8 | F9 | None | Total |
| --- | --- | --- | --- | --- | --- | --- | --- | --- | --- | --- | --- | --- |
| Persons | 5 | 6 | 12 | 26 | 22 | 2 | 0 | 2 | 0 | 1 | 34 | 110 |
| Frequency (%) | 4.6 | 5.5 | 10.9 | 23.6 | 20 | 1.8 | 0 | 1.8 | 0 | 0.9 | 30.9 | 100 |

F0-9: classification of comorbid psychiatric disorders in the International Statistical Classification of Diseases and Related Health Problems 10th Revision (ICD-10).

**Supplementary Table 2. Dose equivalents of psychotropic medication classes.**

| aripiprazole | 4mg | olanzapine | 2.5mg |
| --- | --- | --- | --- |
| blonanserin | 4mg | paliperidone | 1.5mg |
| bromperidol | 2mg | perospirone | 8mg |
| chlorpromazine | 100mg | quetiapine | 66mg |
| clocapramine | 40mg | risperidone | 1mg |
| clozapine | 50mg | spiperone | 1mg |
| haloperidol | 2mg | sulpiride | 200mg |
| levomepromazine | 100mg | zotepine | 66mg |

Antipsychotic dose equivalents to 100mg of chlorpromazine (Inada & Inagaki 2015).

| amitriptyline | 150mg | lofepramine | 150mg | sertraline | 100mg |
| --- | --- | --- | --- | --- | --- |
| amoxapine | 150mg | maprotiline | 150mg | setiptiline | 6mg |
| clomipramine | 120mg | mianserin | 60mg | sulpiride | 300mg |
| dosulepin | 150mg | milnacipran | 100mg | trazodone | 300mg |
| duloxetine | 30mg | mirtazapine | 30mg | trimipramine | 150mg |
| escitalopram | 20mg | nortriptyline | 75mg |  |  |
| fluvoxamine | 150mg | paroxetine | 40mg |  |  |
| imipramine | 150mg | paroxetine CR | 50mg |  |  |

Antidepressants dose equivalents to 150mg of imipramine (Inada & Inagaki 2015).

| alprazolam | 0.8mg | flutazolam | 15mg |
| --- | --- | --- | --- |
| bromazepam | 2.5mg | flutoprazepam | 1.67mg |
| clobazam | 10mg | loflazepate | 1.67mg |
| clonazepam | 0.25mg | lorazepam | 1.2mg |
| clotiazepam | 10mg | medazepam | 10mg |
| diazepam | 5mg | mexazolam | 1.67mg |
| etizolam | 1.5mg | oxazolam | 20mg |
| fludiazepam | 0.5mg | tofisopam | 125mg |

Benzodiazepine dose equivalents to 5mg of diazepam (Inada & Inagaki 2015).
